# Exploring dementia‐related stigma in conversation between a racialized person living with dementia and their family care partner

**DOI:** 10.1002/alz70858_102494

**Published:** 2025-12-25

**Authors:** George Philip, Marie Y. Savundranayagam, Anita Kothari, Joseph B Orange

**Affiliations:** ^1^ Western University, London, ON, Canada

## Abstract

**Background:**

Dementia‐related stigma is known to perpetuate negative stereotypes, shame, embarrassment, and isolation. This hinders help‐seeking behaviour, resulting in a delayed diagnosis and caregiver stress. These stigmatizing perceptions are profound among racialized persons living with dementia (PLWD) and their care partners. However, little is known of how stigma presents itself in natural, everyday conversation among racialized individuals. Understanding these interactions is critical to uncovering ways stigma is experienced. This study aimed to explore dementia‐related stigma through naturalistic conversations between a racialized PLWD and their family care partner.

**Method:**

Analysis of conversation using audio recordings from naturalistic conversations between racialized Canadians living with dementia and their family care partners was performed. This study used conversation data from the Canadian Consortium on Neurodegeneration in Aging (CCNA), which has collected the largest dataset of interactions between persons living with cognitive impairment and their family care partners across Canada. A sub‐sample of dyads involving racialized PLWD (*n* = 25) from 145 audio recordings was analyzed. Each recorded conversation was approximately 20 minutes in length and focused on five racialized communities (i.e., Black, Hispanic/Latin, South Asian, East Asian, Middle Eastern). Codes were extracted from transcripts and were organized into themes and sub‐themes to identify dementia‐related stigma.

**Result:**

Three overarching themes were generated: 1) Negative Perceptions of Dementia & Aging, with sub‐themes of negative stereotypes and ageism; 2) Family Burden, with sub‐themes of stress on family and family making decisions; and 3) Evaluating memory through testing with sub‐themes of testing with numbers and testing social history.

**Conclusion:**

This study is the first to examine dementia‐related stigma in daily conversation between a racialized PLWD and their family care partner. Dementia‐related stigma between family is often not overt, direct, or intentional in everyday conversation. It provides a deeper understanding of how stigma is perpetuated in everyday language. Future studies will compare dementia‐related stigma using an equivalent subsample of CCNA dyads involving white PLWD and their family care partner.

